# Efficient Adsorption of Nitrogen and Phosphorus in Wastewater by Biochar

**DOI:** 10.3390/molecules29051005

**Published:** 2024-02-26

**Authors:** Xichang Wu, Wenxuan Quan, Qi Chen, Wei Gong, Anping Wang

**Affiliations:** 1 Key Laboratory for Information System of Mountainous Area and Protection of Ecological Environment of Guizhou Province, Guizhou Normal University, Guiyang 550025, China; wuxichang@gznu.edu.cn; 2 School of Materials and Architectural Engineering, Guizhou Normal University, Guiyang 550025, China; qichen@gznu.edu.cn (Q.C.); gongw@gznu.edu.cn (W.G.)

**Keywords:** eutrophication, biochar, adsorption, nitrogen, phosphorus

## Abstract

Nitrogen and phosphorus play essential roles in ecosystems and organisms. However, with the development of industry and agriculture in recent years, excessive N and P have flowed into water bodies, leading to eutrophication, algal proliferation, and red tides, which are harmful to aquatic organisms. Biochar has a high specific surface area, abundant functional groups, and porous structure, which can effectively adsorb nitrogen and phosphorus in water, thus reducing environmental pollution, achieving the reusability of elements. This article provides an overview of the preparation of biochar, modification methods of biochar, advancements in the adsorption of nitrogen and phosphorus by biochar, factors influencing the adsorption of nitrogen and phosphorus in water by biochar, as well as reusability and adsorption mechanisms. Furthermore, the difficulties encountered and future research directions regarding the adsorption of nitrogen and phosphorus by biochar were proposed, providing references for the future application of biochar in nitrogen and phosphorus adsorption.

## 1. Introduction

With the rapid development of the economy, the contradiction between environmental protection and economic growth has caused the general deterioration of water quality in China [1], and eutrophication of water bodies is one of the ecological problems. The word “eutrophication” comes from the Greek word Europhos [2], which means good nutrition. It is considered an environmental challenge and harms aquatic ecosystems and drinking water needed by human life. Recently, it has become a global environmental problem [3], usually caused by the excessive supply of nutrients such as nitrogen and phosphorus in water [4]. It can spread algae and endanger the safety of drinking water and the biodiversity of aquatic ecosystems [5], thus endangering the health of fish and even human beings. Therefore, the removal of nitrogen and phosphorus in water is important.

Biochar is a carbon-rich substance formed by the thermal decomposition of biomass [6]. It has ample surface area, and its porous structure enables it to remove various pollutants, such as heavy metals, nitrogen, and phosphorus [7]. In the aspect of nitrogen and phosphorus removal, researchers utilized FeCl_3_ impregnated corn straw powder to prepare biochar, forming a layer of iron oxide on the surface of biochar, which altered the surface morphology and internal crystal structure of biochar. According to the Langmuir model, the adsorption capacity for phosphorus reached over 90 mg/g [8]. Meanwhile, Li et al. [9] also utilized corn stalks as raw materials to prepare modified biochar Ca/MgBC by adding chemical reagents. This biochar demonstrated effective adsorption of nitrogen and phosphorus, with adsorption capacities of 177.25 mg/g and 253.95 mg/g, respectively. The main adsorption mechanism was the formation of struvite crystallization. Researchers have also developed a more efficient biochar adsorbent material (ESBC) using corn stalks and eggshells, with results showing that ESBC has a maximum phosphorus adsorption capacity of 557 mg/g [10]. Biochar has the potential for extensive future applications due to its highly efficient adsorption characteristics. In agriculture, phosphate-saturated biochar shows excellent potential as an environmentally friendly phosphate fertilizer [11]. Nitrogen and phosphorus are essential elements for plant growth. The recovery of nitrogen and phosphorus reduces the pollutions’ harm to the environment and they can be recycled in agriculture, increasing the utilization efficiency. Experimental research shows that biochar with adsorbed nitrogen and phosphorus can promote the growth of mung beans in terms of plant height, fresh weight, dry weight, and chlorophyll content, which can somewhat alleviate the pressure of using fertilizers [12]. Currently, the removal methods for nitrogen and phosphorus in water mainly include chemical, physical, and biological processes [13]. Among them, adsorption is considered one of the most valuable methods to remove ammonium and phosphate from water because of its low cost and high removal efficiency [14]. Among adsorption materials, biochar raw materials have the advantages of comprehensive sources, standard environmental and economic costs [15], high specific surface area, high porosity, and a wealth of functional groups [16]. Therefore, using biochar to remove nitrogen and phosphorus from wastewater has good application prospects.

## 2. Preparation of Biochar

### 2.1. Preparation of Original Biochar

**Figure 1:**
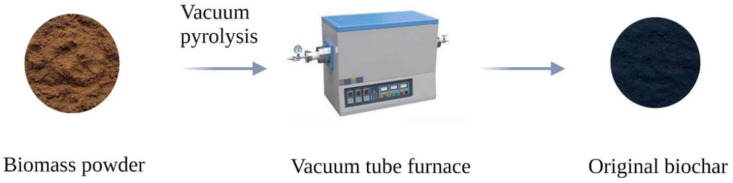
Preparation process of original biochar.

Primitive biochar is obtained with pyrolysis of biomass raw materials without chemical treatment under high temperatures and oxygen deficiency. As shown in Figure 1, the biomass feedstock powder is subjected to high-temperature pyrolysis in a muffle furnace or tubular furnace, resulting in the formation of raw biochar under a nitrogen-rich environment. The preparation of original biochar does not need chemical reagents for modification or other treatment, and the list of sources of biomass is extensive, which has the advantages of economy and simple preparation. For example, Liu et al. [17] directly pyrolyzed pomelo peel powder to obtain biochar “PB”. The preparation process is relatively simple and results regarding the specific surface area and total pore volume were much smaller than those of treated grapefruit peel powder. Similarly, Chen et al. [18] cut corn and rice straw into 5~10 cm pieces, placed them in a muffle furnace, and pyrolyzed them at 500 °C for 2 h to obtain biochar. In addition to the above three raw materials, other researchers have successfully prepared original biochar by direct pyrolysis of peanut shell [19], pine wood [20], wheat straw [21], rape straw [21], potato straw [21], soybean [22], pineapple leaves [23], and sugarcane bagasse [23]. These biochars exhibit excellent adsorption performance, low cost, and are environmentally non-polluting materials, making them a material worth studying and utilizing in environmental remediation.

### 2.2. Preparation of Modified Biochar

#### 2.2.1. Preparation of Biochar by Modification before Pyrolysis

**Figure 2:**
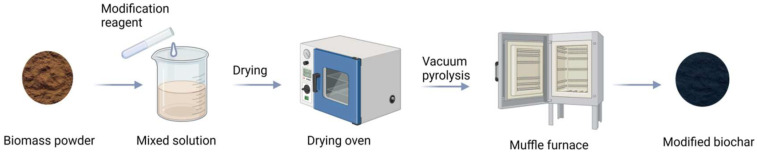
The process of preparing biochar with modification before pyrolysis.

Generally, biomass modified by chemical reagents has more potential to generate biochar with pyrolysis. As shown in Figure 2, the biomass powder reacts with the modifying agent for some time. It is dried in a drying oven and then pyrolyzed at high temperature in a muffle or tube furnace to form modified biochar. For example, Qin et al. [24] mixed poplar with Fe(NO_3_)_3_ and ZnCl_2_, stirred for 24 h, dried the mixture, and finally pyrolyzed it at high temperature in a tube furnace to obtain biochar. The specific surface area of the biochar was as high as 1835.6 m^2^·g^−1^. Liu et al. [25] prepared Fe-biochar. Pretreated peanut shell powder was mixed with FeCl_3_ solution, stirred by magnetic force at 80 °C for 2 h, dried to a constant weight, and pyrolyzed in a high-temperature anaerobic environment to obtain Fe-biochar.

Similarly, the researcher mixed macadamia nut shell powder with K_2_FeO_4_ for some time, dried it, and put it in a tube furnace for high-temperature pyrolysis to obtain modified biochar BC-K_2_FeO_4_. The pore volume reached 0.2176 cm^3^·g^−1^, which is much larger than that of unmodified macadamia nut shell biochar (0.0165 cm^3^·g^−1^) [26]. Currently, most researchers prefer to use this method to prepare modified biochar because modified biochar has high specific surface area and abundant functional groups and adsorption sites, which result in higher adsorption rates for nitrogen and phosphorus.

#### 2.2.2. Preparation of Biochar by Modification after Pyrolysis

**Figure 3:**
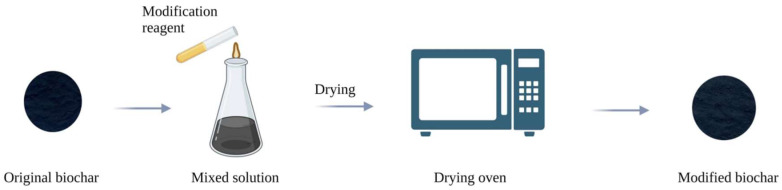
Process of preparing biochar with postpyrolysis modification.

Modification after pyrolysis means that the original biochar reacts with chemical reagents for a certain period, and then the modified biochar is obtained through drying. The preparation process is shown in Figure 3. For example, Jiang et al. [27] directly pyrolyzed Suaeda salsa at 800 °C to generate original biochar 800-SBC, then added a certain amount of original biochar 800-SBC into an FeCl_3_ solution, stirred at constant temperature in a water bath for 3 h, washed with HCl and NaOH solution to neutrality, washed with deionized water, and finally dried in an oven to obtain modified biochar Fe-800SBC. Similarly, Huang et al. [28] prepared the original biochar from biomass, added the original biochar, FeSO_4_·7H_2_O, and MnSO_4_·H_2_O in a plastic conical flask with a weight ratio of 1:2.24:0.7 for modification, and finally stirred, centrifuged, washed and dried to obtain the modified biochar. Xiao et al. [29] mixed the original grapefruit peel biochar with nitric acid according to a specific mass ratio to retain more acidic functional groups and adsorption sites. They obtained modified biochar after stirring, washing, and drying.

## 3. Modification Methods of Biochar

### 3.1. Chemical Modification

Using appropriate chemical reagents to modify biochar can increase its adsorption capacity. Chemical modification can be divided into inorganic modification and organic modification. Common inorganic substances include acids, bases, and salts, while organic compounds include vitamins and polymers. Researchers used FeCl_3_·6H_2_O and MgCl_2_·6H_2_O to co-modify water hyacinth, and found that the modified biochar had a rougher surface compared to the original biochar, with increased surface area and pore volume [30]. Similarly, Xu et al. [31] modified water hyacinth with citric acid and found that the modified biochar contained more oxygen functional groups and exhibited stronger hydrophilicity. However, the surface area and pore volume decreased by 38.6% and 32.6%, respectively. This may be attributed to the residual citric acid trapped in the pores. The influence of inorganic modification and organic modification on biochar is reflected in surface area, total pore volume, average pore size, atomic O/C ratio, and adsorption capacity. As for the extent to which organic compounds alter the characteristics of biochar, it depends on the content and structure of the compound [32]. In most cases, the organic modifications does not improve textural parameters of BC [33]. Fan et al. [34] utilized chitosan for modification and found that the modified biochar exhibited increased O/N content but decreased surface area and pore volume. In conclusion, the chemical modification process is relatively simple and can save time and costs. However, the modification of some organic compounds does not improve the structural parameters of biochar. The use of appropriate chemical reagents to modify biochar can improve its structural parameters, ensuring a greater extent of nitrogen and phosphorus adsorption and reducing environmental hazards. On the other hand, although chemical modification can to some extent enhance the adsorption of nitrogen and phosphorus by biochar, the chemical reagents added during modification may potentially cause secondary pollution to the environment.

### 3.2. Physical Modification

Ball milling and steam activation are the most widely used physical modification methods for biochar. Both methods effectively increase the surface area and the number of internal pores (mainly micropores) of biochar. The modified biochar possesses a higher content of oxygen-containing functional groups, such as carboxyl and hydroxyl groups [35]. Gong et al. [36] placed the raw biochar into a ball mill, with a ball diameter of 3 mm and a rotation speed of 580 r/min. The mass ratio of balls to biochar was set at 8:1, resulting in the successful production of modified biochar. The modified biochar exhibited a surface area and pore volume 6.94 times and 5.25 times higher than that of the raw biochar, respectively. In addition, the aperture and carbon content have also increased. Physical modification can also be achieved by introducing gases (such as CO_2_ and NH_3_) during the pyrolysis of biochar. These gases react with biochar to achieve the desired modification, or the pre-pyrolyzed raw biochar can be modified by exposure to ultraviolet (UV) radiation. Zhang et al. uniformly spread the raw biochar on a glass petri dish, then exposed it to a 250 W UV lamp with a wavelength of 365 nm at a distance of 40 mm for 16 h. After cooling to room temperature, the characterized results showed the presence of irregular pores on the surface of the biochar after UV exposure, with increased surface area and average pore size [37]. In addition, biomass can also be pyrolyzed under nitrogen atmosphere, followed by activation with CO_2_ or water vapor to prepare biochar. The organic compounds on the surface of biochar activated by steam are degraded, resulting in the formation of a porous structure. The surface area increased from the original 0.22 m^2^/g to 514 m^2^/g [38]. The appearance of a porous structure increases the surface area of the biochar. Physical modification can provide the biochar with more functional groups, surface area, and pore volume, which are advantageous for the adsorption of nitrogen and phosphorus by the biochar. Furthermore, modification of biochar using ball milling and steam activation does not cause secondary pollution to the environment.

### 3.3. Biological Modification

Microorganisms can immobilize amino, carboxyl, hydroxyl groups, and other functional groups onto the surface of biochar, thereby enhancing its adsorption efficiency. However, the bio-modification of biochar is a complex process, and the activity of microorganisms can be influenced by environmental factors such as temperature [39]. Generally speaking, there are three techniques for immobilizing bacteria on biochar: growth on biochar, adsorption after microbial growth, and preactivation before immobilization [40]. However, these methods may incur higher costs [41], and compared to chemical and physical modifications, the bioprocessing for biologically modified materials is more complex. Tu et al. [42] successfully immobilized bacteria (*Pseudomonas* sp. NT-2) onto corn straw biochar. Initially, NT-2 was cultured in a growth medium at a constant temperature. Subsequently, the biochar was mixed with NT-2 in a ratio of 1:3 (*w*/*v*) and maintained for 8 h. Finally, the NT-2-loaded biochar was freeze-dried, and scanning electron microscopy revealed that NT-2 exhibited excellent attachment to the surface and pores of the biochar. Researchers have also employed bacterial strains (Bacillus megaterium-168 and Bacillus subtilis-J) for secondary modification of Fe_3_O_4_-loaded corn straw biochar, and it was found that the modified biochar exhibited a significantly larger surface area and pore volume compared to the unmodified biochar, thereby increasing the availability of more adsorption sites [43]. In addition, biological modification can also increase the oxygen-containing functional groups of biochar [41]. On the other hand, the high specific surface area of biochar provides suitable habitats for microorganisms, reducing the impact of environmental factors on them. However, the physicochemical properties of biochar itself also affect the survival rate of microorganisms.

## 4. Study on the Adsorption of Nitrogen and Phosphorus by Biochar

### 4.1. Adsorption of Nitrogen by Biochar

Nitrogen is essential for plant growth, but excessive nitrogen will also pollute the environment. Recently, the adsorption of nitrogen by biochar has achieved good results. On the original biochar, Cheng et al. [3] used the invasive plant Eupatorium adenophorum to prepare biochar at 300 °C, and the maximum adsorption capacity of ammonium was 1.909 mg/g, which not only solved the ecological invasion problem of Eupatorium adenophorum but also realized the adsorption of nitrogen. Other researchers used rice husk to prepare biochar, and after pyrolysis at 300 °C for 2 h, they carried out adsorption experiments on ammonium nitrogen. The reaction time was 1~3 min, and the maximum adsorption capacity reached 32 mg/g [44]. Compared with Eupatorium adenophorum, rice husk biochar has a higher ammonium adsorption capacity without reagent modification. Although the original biochar can effectively adsorb nitrogen, we must prepare more efficient adsorbents in an environment with severe pollution. In addition, biochar can be modified by chemical reagents or other composite materials to achieve or exceed the effect of traditional adsorbents [45]. For example, the maximum adsorption capacity of biochar made by soaking coffee grounds in KOH solution for 2 h is 51.52 mg/g [46]. Li et al. [47] used walnut shells as raw material and FeCl_3_·6H_2_O and dicyandiamide as modifying reagents. They obtained Fe/N@BC using pyrolysis in a tube furnace at 600 °C for 2 h and passing through an 80~120 mesh sieve. As a result, Fe/N@BC has a large specific surface area, rich pore structure, and many adsorption sites, and its adsorption effect on ammonium is better, with the maximum adsorption capacity reaching 111.87 mg/g. When the raw materials are the same, the adsorption efficiency of ammonium nitrogen can be increased by modifying them with appropriate reagents. Tan et al. [48] compared the adsorption of original biochar (BC) and modified biochar (NBC, MBC, NMBC) prepared from corn straw. After testing, the adsorption capacity of ammonium nitrogen ranking was NMBC > MBC > NBC > BC. It can be seen that the adsorption capacity of ammonium nitrogen by biochar is different when modified with other reagents. Table 1 summarizes the progress of nitrogen adsorption by biochar, indicating variations in nitrogen adsorption capacity depending on different factors such as feedstock and preparation methods.

### 4.2. Adsorption of Phosphorus by Biochar

In life, industrial and agricultural wastewater carries phosphorus and flows into lakes, rivers, and other water bodies [56]. Excessive phosphorus released into water bodies will lead to algae growth, eutrophication, water quality decline, and other severe environmental problems [57], which is a significant motivation for phosphorus recovery. Some researchers used coffee powder as a raw material, and Fe_12_LaO_19_@BC was obtained by coprecipitation of iron and lanthanum, mainly forming lanthanum phosphate and iron phosphate to adsorb phosphorus [58]. In addition, phosphorus can also be combined with biochar through electrostatic attraction and complexation. For example, the biochar BMCa@BC prepared by Ai et al. [59] with CaO and corn stalk as raw materials has a high phosphorus recovery efficiency, and the maximum adsorption capacity of phosphorus reached 329 mg/g, which provides a new method for phosphorus recovery. Before and after biochar modification, researchers prepared biochar from biomass longleaf pine wood shavings, red oak, and hard maple sawdust. It was found that the adsorption capacity of biochar modified by MgCl_2_ was 11 times that of the original biochar [60], significantly improving the adsorption capacity of phosphorus. Wang et al. [61] prepared the biochar Ca-pmsBC750N from the sludge of a paper mill, which can also effectively recover phosphorus and mainly forms CaHPO_4_ and Ca_5_(PO_4_)_3_(OH), which is a process dominated by chemical adsorption. On the other hand, Zhang et al. [62] fermented sludge anaerobically and then put it into a tubular furnace for high-temperature pyrolysis. The produced biochar (ES600) can effectively remove phosphorus, with a removal efficiency of 98.6%, and even after three desorption cycles, it can still maintain a high removal rate.

In contrast, the biochar produced by the team mixing CaCl_2_ into sludge had a higher phosphorus adsorption capacity, reaching 227.14 mg/g [63]. The efficient conversion road of waste material reuse was realized. Table 2 summarizes the progress of biochar in terms of phosphorus adsorption. It can be observed that pyrolysis temperature, modification reagents, and feedstock all influence the adsorption efficiency of biochar for phosphorus.

## 5. Factors Affecting Adsorption of Nitrogen and Phosphorus by Biochar

**Figure 4:**
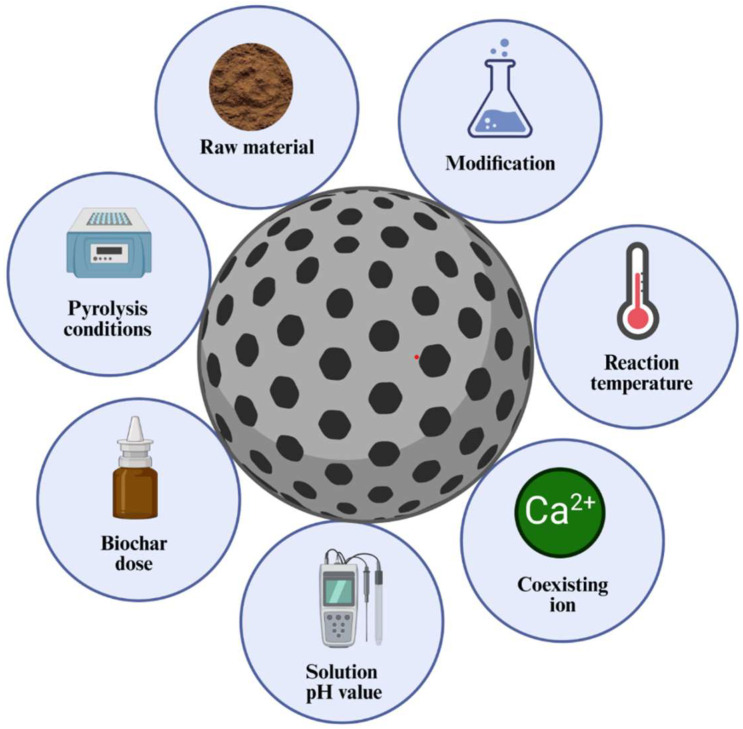
Analysis of influencing factors on the adsorption of nitrogen and phosphorus in water by biochar.

The adsorption effects of biochar on nitrogen and phosphorus mainly include biomass raw materials, modification of biochar, pyrolysis conditions, pH value of the solution, coexisting ions, reaction temperature, and dosage, as shown in Figure 4.

### 5.1. Influence of Preparation Conditions of Biochar

#### 5.1.1. Influence of Raw Materials

Different biomass raw materials have different structures, and the chemical element content [74], specific surface area [75], ash content [76], and functional groups [74] contained in biochar after pyrolysis are also different, which will further affect the adsorption effects of biochar. Typical raw materials are corn [18,69], straw [77,78], peanut hull [19], wheat straw [21], rape straw [21], animal manure [79], and some agricultural wastes [80,81,82], which can be anaerobic at high temperatures. Researchers [83] used sewage sludge and walnut shells to prepare the original biochar and found that the carbon content and specific surface area of sewage sludge biochar (SBC) were much lower than those of walnut shell biochar (BC), but it contained more metal elements than walnut shell biochar. The pore size distribution of BC is concentrated in micropores (<2 nm) and mesopores (6–11 nm), while the pore size distribution of SBC is concentrated in micropores (<2 nm) and macropores (10–20 nm). In NH_4_^+^-N adsorption, the adsorption capacities of SBC and BC were 0.62 and 1.58 mg/g, respectively, which may be caused by the different surface areas of the two biochars. In the PO_4_^3−^-P adsorption experiment, the adsorption capacities of SBC and BC for PO_4_^3−^-P were 303.49 and 0.65 mg/g, respectively. SBC has a relatively high phosphorus adsorption capacity, which is related to the fact that its metal elements (Ca, Al, Fe, etc.) can precipitate or form a complex with PO_4_^3−^. The results show that the two kinds of original biochar prepared from wood and rice husk are different in specific surface area, total pore volume, pore size, and element content (C, H, O, N, etc.); the micropore volume of wood biochar (0.1123 mL/g) is larger than that of rice husk biochar (0.0044 mL/g); and wood biochar has higher efficiency in removing NH_4_^+^-N [76]. Other studies have shown that the surface of the original biochar prepared from mimosa is smooth and uniform, and there are many small holes. The adsorption capacity of phosphorus was 5.1 mg/g [84]. It can be seen that the original biochar prepared from different biomasses shows different adsorption of nitrogen and phosphorus, which the characteristics of the biochar itself may cause. However, how to screen biochar raw materials with high adsorption capacity for nitrogen and phosphorus needs further study.

#### 5.1.2. Influence of Chemical Modification

**Figure 5:**
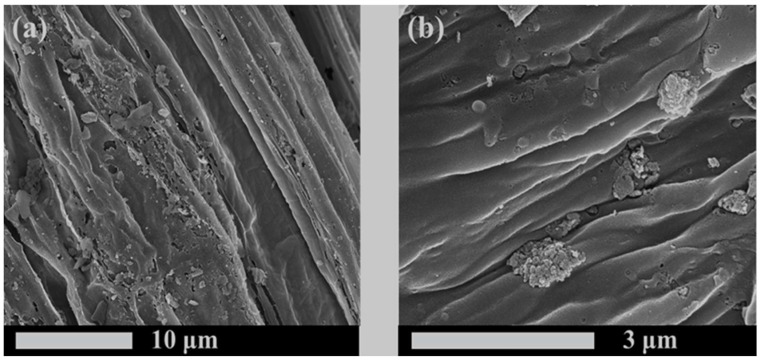
(**a**) SEM of BC, 10,000×, (**b**) SEM of BC, 50,000×. Reprinted with permission from Ref. [91]. Copyright 2023 Copyright Hui Hu, Min Gao, Tian Wang, Lei Jiang.

**Figure 6:**
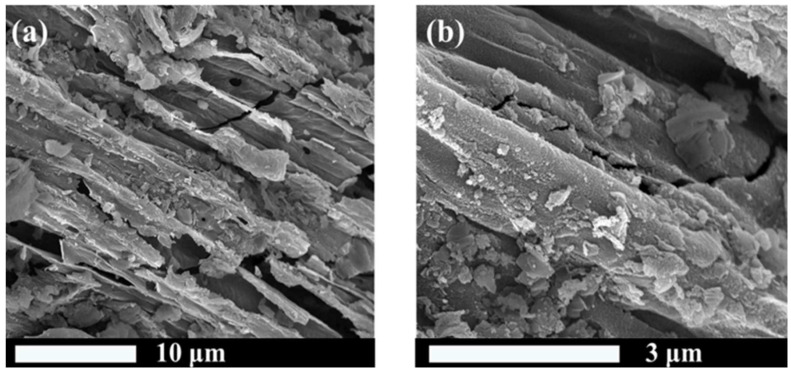
(**a**) SEM of MBC, 10,000×, (**b**) SEM of MBC, 50,000×. Reprinted with permission from Ref. [91]. Copyright 2023 Copyright Hui Hu, Min Gao, Tian Wang, Lei Jiang.

**Figure 7:**
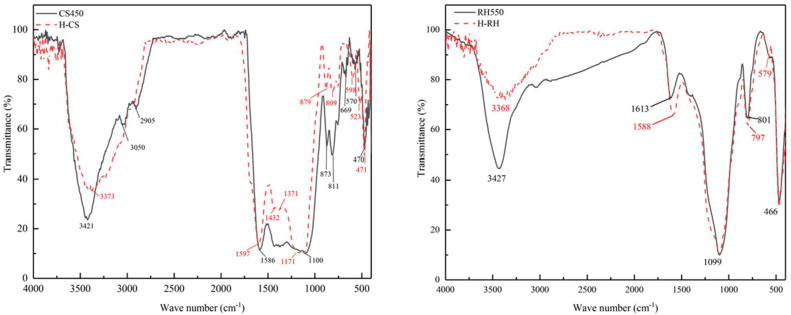
FTIR spectra original biochar (CS450 and RH550) and modified biochar (H-CS and H-RH). Reprinted with permission from Ref. [93]. Copyright 2021 Copyright Mei Chen, Fang Wang, De-li Zhang, Wei-ming Yi, Yi Liu.

At present, there are many studies on the modification of biochar. The common modification reagents are Ca(OH)_2_ [85], MgCl_2_ [86], FeCl_3_ [87], Mg(NO_3_)_2_ [88], CaCl_2_ [89], and KOH [90]. It was found that the biochar impregnated with FeCl_3_ increased the surface charge and the content of reducing oxygen-containing functional groups (C-O) [87]. In addition, compared to the original biochar (Figure 5a,b), the biochar modified with MgCl_2_·6H_2_O (Figure 6a,b) exhibits a rougher heterogeneous surface and smaller particles. Additionally, the ash content and pH value are both increased [91]. The results show that the biochar pretreated with KMnO_4_ increases the specific surface area and pore volume, changes the types, elements, and crystal mineral composition of surface functional groups; enriches the pore structure; and forms a carbon defect structure in biochar [92]. Biochar modified with chemical reagents can increase the adsorption capacity of nitrogen and phosphorus. As reported by Zhuo et al. [71], the original biochar has a smooth surface with regular and straight channels. After being modified by CaCl_2_, the surface is roughly covered by crystalline particles; at the same time, pores are formed, and the surface area and total pore volume are also increased. The modified biochar has a stronger adsorption capacity for phosphate removal. Moreover, research has shown that the use of H_2_SO_4_ for acid modification of corn stalks and rice husks resulted in an increase in the number of acidic oxygen-containing functional groups and enhanced aromaticity in the modified biochar (Figure 7). After modification, the peak of the O-H stretching vibration at about 3400 cm^−1^ decreased. And the peak at 2950–2834 cm^−1^, corresponding to aliphatic symmetrical and asymmetric C-H bonds, disappeared. The peaks of C=C and C=O on the aromatic carbon structure at 1612–1585 cm^−1^ were enhanced; this indicates an increased aromaticity of the modified biochar. It also showed a better ammonium adsorption effect than the original biochar in both pure solution and mixed solution. The reason may be that the modification removed ash, opened more blocked pores, and provided more adsorption sites, thus enhancing the adsorption capacity of ammonium [93]. As shown in Table 3 and Table 4, the comparison of adsorption of nitrogen and phosphorus between modified biochar and original biochar is summarized. The specific surface area and total pore volume of the modified biochar changed, and most modified biochars had greater adsorption capacities for nitrogen and phosphorus. At present, there are many studies on the effect of pretreatments, but the reasons have not been discussed in depth, and there is no relevant discussion on whether they will have adverse effects on the environment while improving the adsorption effects [94].

#### 5.1.3. Influence of Pyrolysis Conditions

Pyrolysis temperature is an important factor affecting the adsorption of nitrogen and phosphorus by biochar. Some researchers found [98] that with the pyrolysis temperature increasing from 400 °C to 600 °C, the surface area and total pore volume showed an upward trend because at higher pyrolysis temperatures, the loss of volatile substances would promote the formation of more micropores, which led to an increase in pore volume and specific surface area [99]. However, there were also cases where the surface area and pore volume increased or decreased irregularly with increasing temperature [100]. Moreover, with increasing temperature, the alkalinity of biochar will be enhanced, which may be due to the decrease in -COOH and -OH functional groups, while an increase in basic anions (CO_3_^2−^ and HCO_3_^−^) will be produced with increasing pyrolysis temperature, resulting in an increase in the pH value of biochar [101]. Chun et al. [102] also found that the O and H functional groups on the surface of biochar produced at higher pyrolysis temperatures were removed, resulting in lower acidity and increased aromaticity and hydrophobicity. In addition, unsaturated carbon is gradually transformed into stable carbon with increasing pyrolysis temperature, the condensation degree of the aromatic structure is gradually improved, and the stability is enhanced [103]. In terms of nitrogen adsorption, Xu et al. [104] prepared biochar from four raw materials: rice straw, reed, eggshell, and sawdust. Each raw material was pyrolyzed at 300 °C, 500 °C, and 700 °C. The results showed that the biochar prepared by pyrolysis at 700 °C had the smallest adsorption capacity of NH_4_^+^-N, and the zeta potential of biochar pyrolyzed at 700 °C was negative. Yuan et al. [105] also identified similar findings. In the adsorption of phosphorus, researchers used sludge to prepare biochar at temperatures of 300 °C, 400 °C, 500 °C, 600 °C, 700 °C, and 750 °C. The results showed that the removal efficiency of phosphorus gradually increased for biochar prepared at temperatures ranging from 300 °C to 700 °C. However, it significantly decreased when the pyrolysis temperature reached 750 °C. Biochar formed at 600 °C or lower temperatures exhibited very low phosphorus removal rates [106]. At the same time, pyrolysis time and heating rate can affect the stability of biochar [107]. Researchers found that the carbon yield decreases by 2% when the pyrolysis time is extended from 30 min to 120 min [52]. However, the stability of biochar improves as the pyrolysis time increases from 20 min to 80 min [108], and prolonged pyrolysis can enhance the carbonization degree of biochar, making it less susceptible to microbial attack [109]. As the pyrolysis time increases, the surface area and pore volume of biochar also increase [110], providing more adsorption sites for nitrogen and phosphorus. However, the influence of pyrolysis time on the properties of biochar is mostly focused on the surface and has not been thoroughly investigated. Future research can be directed towards exploring this aspect [94]. In addition, there are reports suggesting that an increased heating rate contributes to the formation of more micropores in biochar, but the biochar yield gradually decreases [111]. The surface area and pore volume also decrease with an increase in heating rate. This is because at lower heating rates, the pyrolysis products have sufficient time to be emitted, whereas at faster heating rates, the diffusion time for the pyrolysis products to escape is shorter, resulting in their accumulation within the pores and consequently reducing the surface area and pore volume [112]. Therefore, it is necessary to coordinate the values of pyrolysis temperature, pyrolysis time, and heating rate during the pyrolysis of biochar in order to maximize the production of biochar with high adsorption capacity for nitrogen and phosphorus, thereby exerting its environmental protection effects.

### 5.2. Environmental Factors Affecting Nitrogen and Phosphorus Adsorption by Biochar

#### 5.2.1. pH of the Solution

**Figure 8:**
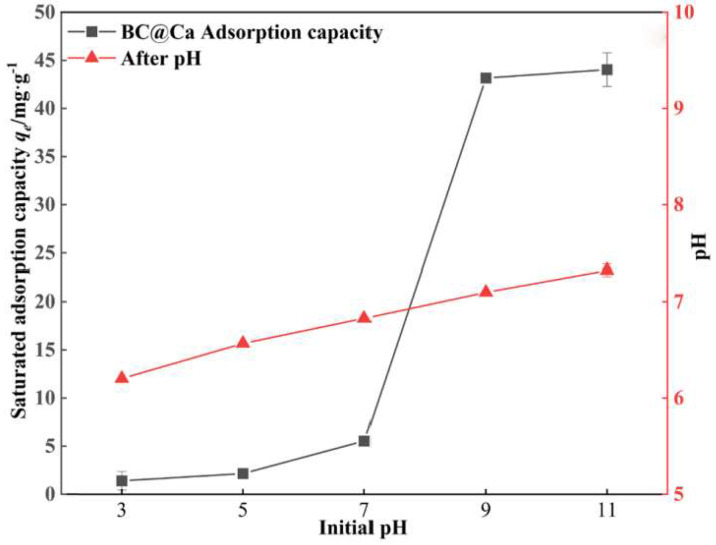
Effect of different initial pH values on the adsorption of P by biochar (BC@Ca). Reprinted with permission from Ref. [117]. Copyright 2022 Copyright Zhihao Chen, Yonghong Wu, Yingping Huang, Linxu Song, Hongfeng Chen, Shijiang Zhu, Cilai Tang.

The pH value can often affect the adsorption effect of adsorbents in solution. Moreover, the alkalinity of biochar impregnated with magnesium is strong. Even if the initial pH is 3, it may increase to 9 [113], which will affect the adsorption capacity of biochar. Because phosphate exists in different forms in different pH environments, the adsorption capacity of biochar for phosphate will also change. Li et al. [114] found that the adsorption capacity of phosphate on biochar was the largest at pH = 9, and it regularly increased with increasing pH from 4 to 9, which may be due to ligand exchange, which made CO_3_^2−^ fall off the biochar into solution. Similarly, foreign researchers found that biochar adsorbed phosphorus from solution by forming magnesium phosphate residue, and the adsorption amount increased with increasing pH [115]. However, some researchers found that phosphorus adsorption decreased with increased pH [116]. As shown in Figure 8, when the pH of Ca-loaded biochar (BC@Ca) prepared by Chen et al. [117] increased from 3 to 11, the adsorption capacity of phosphorus increased, and the adsorption capacity increased obviously from 7 to 9. The reason is that H_2_PO_4_^−^ does not easily precipitate with Ca^2+^ in a lower pH solution, and it is beneficial to form hydroxycarbonate in a high pH solution. Thus, phosphorus can be effectively removed. However, the Zn-loaded biochar (BC@Zn) they prepared had the opposite effect. When the pH of the solution was 3, the maximum adsorption effect was achieved, and when the pH was greater than 3, the adsorption capacity decreased. It can be seen that it is possible to make full use of its advantages when modifying biochar in the future to produce adsorbents with high adsorption capacity in both acidic and alkaline environments.

**Figure 9:**
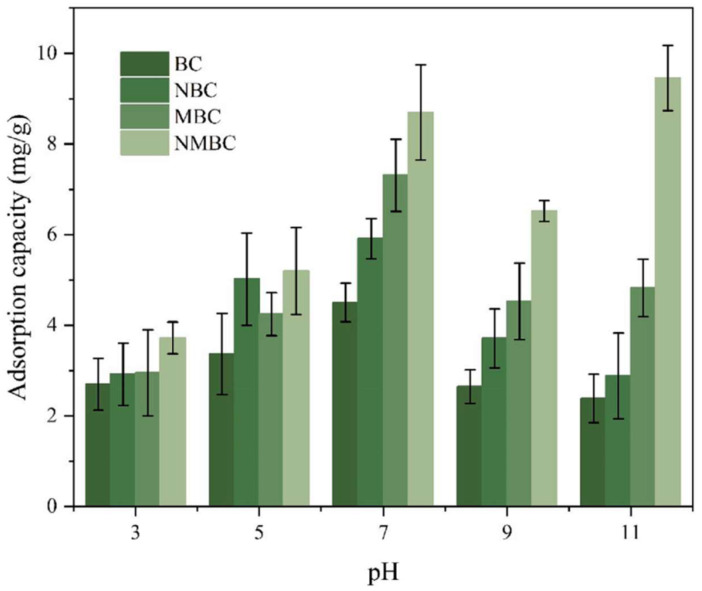
The impact of different pH values on the adsorption of NH_4_^+^ by four types of biochar (BC, NBC, MBC, and NMBC). Reprinted with permission from Ref. [48]. Copyright 2023 Copyright Meitao Tan, Yanqi Li, Daocai Chi, Qi Wu.

This will affect the adsorption of NH_4_^+^ in strong acid and alkali environments. In an alkaline solution, NH_4_^+^ will react with OH^−^ to reduce the concentration of NH_4_^+^ [118]. Under acidic conditions, many H^+^ will compete with NH_4_^+^ for adsorption sites, and researchers have also proven this view through experiments [119]. Therefore, most people think that a weakly alkaline environment is more conducive to the adsorption of NH_4_^+^ [62]. In the adsorption process of NH_4_^+^ by general biochar, the adsorption capacity first increases and then decreases with increasing pH value. The adsorption capacity of the four biochars prepared by Wang et al. [120] gradually increased in solutions with pH ranging from 3 to 6, while it decreased in the pH range of 6 to 10. However, other studies [48] have found that the removal efficiency of NH_4_^+^ increases at pH values of 9 to 11, as shown in Figure 9. The adsorption capacity of biochar MBC and biochar NMBC at pH 11 is higher than that at pH 9, which can be attributed to the volatilization of NH_4_^+^ in gaseous form, leading to “false” adsorption of NH_4_^+^. Another reason is related to the pHpzc (point of zero charge) of the biochar. As the pH of the solution increases, the electrostatic repulsion between NH_4_^+^ and biochar weakens, resulting in an increase in NH_4_^+^ adsorption capacity. In addition, in the lower pH solution, the dissolution of insoluble crystalline minerals in biochar will increase, which will release some cations (K^+^, Ca^2+^, and Mg^2+^), and these cations will also compete with NH_4_^+^ for adsorption [121], thus reducing the adsorption capacity of NH_4_^+^ by the adsorbent.

#### 5.2.2. Coexisting Ions

**Figure 10:**
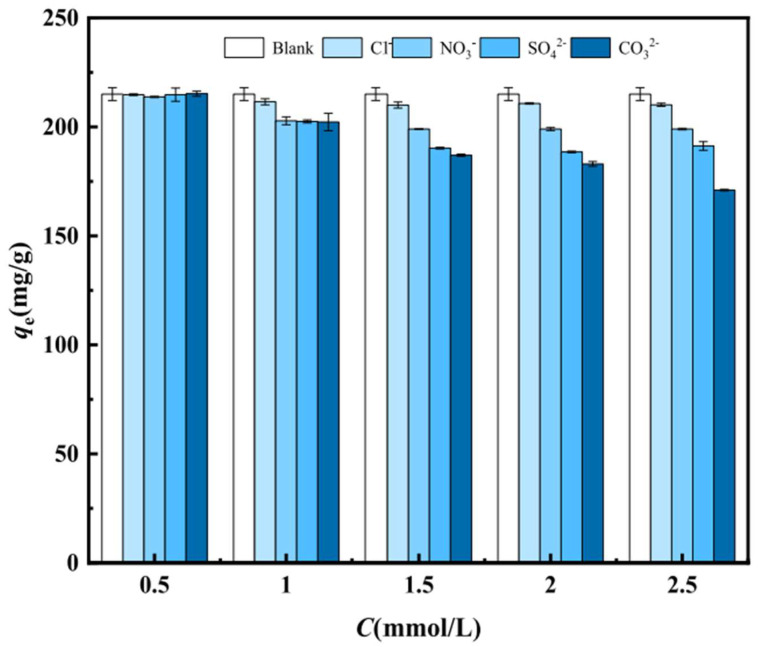
Effect of coexisting anions on the phosphate adsorption capacity of N/CaO/BC. Reprinted with permission from Ref. [122]. Copyright 2022 Copyright Yunwen Liao, Si Chen, Qian Zheng, Bingyuan Huang, Juan Zhang, Hongquan Fu, Hejun Gao.

In the adsorption test of actual pollutants, other ions often affect the target pollutants. The influence of coexisting ions on phosphorus adsorption can be summarized into two aspects: (1) precipitation and (2) synergy. Some researchers found that HCO_3_^−^ in the solution will weaken the removal of phosphorus by the adsorbent because HCO_3_^−^ will first release Ca^2+^ with the biochar to form CaCO_3_ precipitates, while CaHPO_4_ will be created after precipitation (the Ksp of CaCO_3_ and CaHPO_4_ is 3.4 × 10^−9^ and 1.0 × 10^−7^, respectively), so HCO_3_^−^ harms the removal of phosphorus from the biochar [59]. In addition, SO_4_^2−^ will also reduce the adsorption of phosphorus by biochar, and SO_4_^2−^ is readily adsorbed by biochar (CaBC800) to form CaSO_4_ precipitate, thus decreasing the adsorption efficiency by 32.94% [71]. According to the research results of Liao et al. [122], as shown in Figure 10, the order of influence of Cl^−^, NO_3_^−^, SO_4_^2−^, and CO_3_^2−^ on phosphate adsorption of biochar (N/CaO/BC) is CO_3_^2−^ > SO_4_^2−^ > NO_3_^−^ > Cl^−^, which also forms CaCO_3_ and CaSO_4_ precipitates. When conditions permit, adding some ions to the solution will increase the phosphate adsorption capacity of biochar. Liu et al. [73] found that NH_4_^+^ in solution can promote phosphate adsorption, and the effect increases with increasing NH_4_^+^ concentration. When the molar ratio of NH_4_^+^ to phosphorus is 1:1, the efficiency of phosphorus removal is close to 100%, which may be due to the precipitation of NH_4_^+^ and hydrogen phosphate, thus accelerating the reduction of phosphorus. In addition to NH_4_^+^, Cl^−^ has the same effect [69]. Unlike the report of Zhuo et al. [71], another team thinks that adding SO_4_^2−^ can synergistically affect phosphate adsorption, which can enhance the phosphate adsorption capacity [3]. The conclusions reported by the two groups are different because the characteristics of the biochars they prepared are different, which further affects the inconsistent effect of adding SO_4_^2−^. In addition, Ca^2+^ can combine with some ions through ionic bonds. Insoluble salts are formed, which reduces the phosphorus removal efficiency. For example, F^−^ in the solution integrates with Ca^2+^ to create CaF_2_, and the solubility product constant of CaF_2_ (Ksp = 5.3 × 10^−9^) is lower than that of CaHPO_4_ (Ksp = 2.57 × 10^−7^), so F^−^ also affects the removal of phosphorus with the increase in F^−^ concentration from 100 mg/L to 500 mg/L [61]. Phosphorus-containing anions react with metal ions or other cations to form precipitates, thereby removing phosphorus from water. The phosphorus-containing anions and other anions compete for binding sites with metal ions or other cations. When the phosphorus-containing anions first form precipitates with metal ions or other cations, it is advantageous for phosphorus removal. Conversely, it is disadvantageous for phosphorus removal if the phosphorus-containing anions do not form precipitates.

The influence of coexisting ions on nitrogen adsorption is realized by competing with them for adsorption sites, and both anions and cations can compete with them, which eventually leads to a decline in the ammonium adsorption effect of biochar. Wang et al. [118] used NO_3_^−^ as a competitive ion to affect ammonium adsorption. When the concentration of NO_3_^−^ was lower than 50 mg/L, the adsorption capacity of ammonium increased slightly, but in the range of 50~800 mg/L, the adsorption capacity of ammonium continued to decrease, mainly due to ion competition.

**Figure 11:**
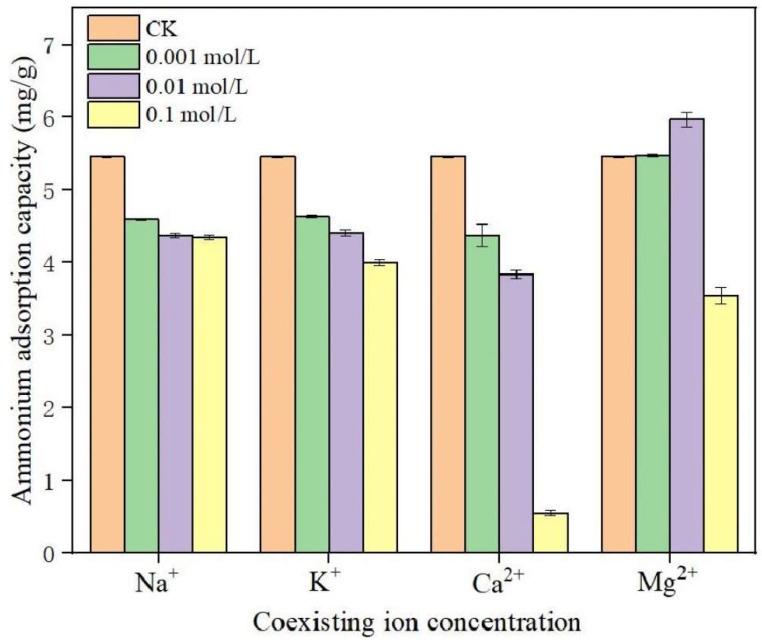
Influence of coexisting ions on the adsorption of ammonium salt. Reprinted with permission from Ref. [12]. Copyright 2022 Copyright Zhipeng Zhao, Bing Wang, Qianwei Feng, Miao Chen, Xueyang Zhang, Ruohan Zhao.

Regarding cations, Fe^3+^ can compete with NH_4_^+^ for adsorption sites on biochar, and eventually, the removal rate of NH_4_^+^ will decrease [3]. However, the ions in the solution are not limited to this. As shown in Figure 11, Zhao et al. [12] found that different concentrations of Ca^2+^, Mg^2+^, K^+^, and Na^+^ can affect the adsorption of ammonium by biochar, and the order of adsorption influence is Ca^2+^ > Mg^2+^ > K^+^ > Na^+^, but adding 0.001 and 0.01 mol/L Mg^2+^ can promote the adsorption of ammonium. The reason is that a low concentration of Mg^2+^ will form a MgNH_4_PO_4_·6H_2_O precipitate with HPO_4_^2−^ and NH_4_^+^ in the solution, while when the concentration of Mg^2+^ is too high, a large amount of MgNH_4_PO_4_·6H_2_O precipitate will be formed to cover the surface and pores of the biochar, thus reducing the adsorption capacity of ammonium of its texture and pores. The factors influencing the adsorption of nitrogen and phosphorus by biochar, such as coexisting ions, have not been thoroughly investigated or explored [94]. In future studies, the mechanisms by which coexisting ions affect the removal of nitrogen and phosphorus by biochar can be examined, and a biochar with high adsorption performance can be developed to improve the current situations.

#### 5.2.3. Effect of Reaction Temperature

The reaction temperature is also one factor affecting nitrogen and phosphorus adsorption by biochar. Grasping the reaction temperature well in adsorption can improve the adsorption efficiency of biochar. When Sun et al. [10] explored the influence of temperature on phosphorus adsorption, it was found that with the increase in temperature (temperature gradient was 291 K, 298 K, and 308 K), the phosphorus adsorption capacity increased from 560.3 mg/g to 588.6 mg/g because the reaction enthalpy of the adsorption process changed ΔH > 0 and the adsorption process was endothermic. Increasing the reaction temperature could accelerate the reaction rate. Similarly, when Li et al. [47] raised the reaction temperature, the adsorption capacity of PO_4_^3−^-P was enhanced, and the adsorption capacity of NH_4_^+^-N was improved. Among the thermodynamic parameters, the Gibbs free energy changed ΔG < 0, and the reaction enthalpy changed ΔH > 0, which indicated that the reaction process could be carried out spontaneously. The reaction rate accelerated with increasing temperature. Wang et al. [120] used four kinds of biochars to adsorb NH_4_^+^-N. They found that both ΔG and ΔH were positive values, in which ΔG decreased with increasing temperature, indicating that increasing the reaction temperature was beneficial to the adsorption process. The reaction entropy changed ΔS > 0, which could increase the disorder and freedom of the solid–liquid interface during the adsorption process.

**Figure 12:**
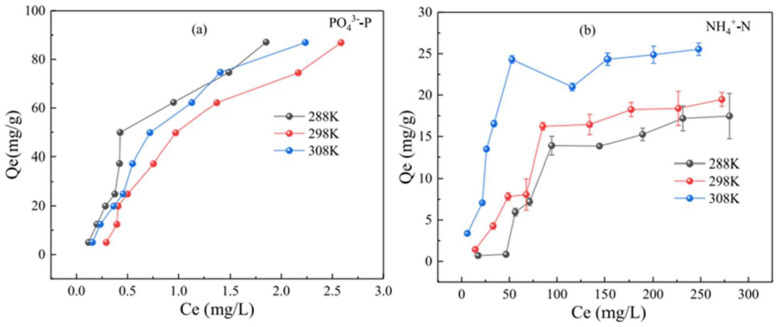
Effect of temperature on the adsorption of PO_4_^3−^-P (**a**) and NH_4_^+^-N (**b**) by CS-MgCBC. Reprinted with permission from Ref. [123]. Copyright 2022 Copyright Lei Li, Qingfeng Chen, Changsheng Zhao, Beibei Guo, Xiaoya Xu, Ting Liu, Lingxi Zhao.

Because some biochars are endothermic in the adsorption process, increasing the reaction temperature can increase the adsorption capacity of nitrogen and phosphorus to some extent, but there are exceptions. Li et al. [123] found that in the endothermic reaction, the increase in temperature may not necessarily increase the adsorption efficiency of PO_4_^3−^-P on biochar. As shown in Figure 12, the adsorption capacity of NH_4_^+^-N increases with increasing temperature, but the adsorption capacity of PO_4_^3−^-P has little effect, and the adsorption capacity of PO_4_^3−^-P at 298 K is less than that at 288 K. Therefore, in the adsorption process of an endothermic reaction, we can not only increase the temperature but also ignore the factors of the material itself, and we can conduct many experiments to determine the best adsorption temperature. In the process of nitrogen and phosphorus adsorption by biochar, most processes are endothermic. Increasing the temperature properly can increase the randomness of the solution interface, which is beneficial to removing NH_4_^+^-N and PO_4_^3−^-P.

#### 5.2.4. Influence of Biochar Dosage

**Figure 13:**
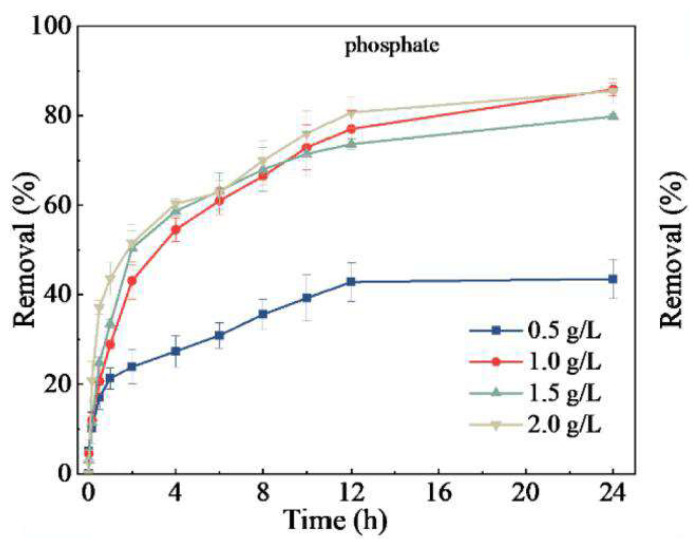
The influence of the dosage of biochar CaBC800 on the efficiency of phosphate removal. Reprinted with permission from Ref. [71]. Copyright 2022 Copyright Sheng-Nan Zhuo, Tian-Chi Dai, Hong-Yu Ren, Bing-Feng Liu.

The amount of adsorbent added is an important factor affecting the adsorption characteristics [124]. In removing nitrogen and phosphorus, increasing the amount of biochar increases the adsorption sites, which is more effective in removing nitrogen and phosphorus. The researchers found that when the biochar dosage was increased, the adsorption capacity of biochar for phosphate first increased and then decreased. At the same time, the adsorption capacity for ammonium nitrogen continued to decline [125], which may be due to the increase in the dosage of biochar, which led to a decrease in the phosphate concentration in the solution and eventually led to a decline in the adsorption capacity [126]. The adsorption capacity and removal rate reached their highest values when a certain amount of biochar was added. As shown in Figure 13, for the influence of biochar dosage on phosphorus adsorption, Zhuo et al. [71] increased the removal efficiency of biochar for phosphate from 0.5 g/L to 1.0 g/L. When the dosage was more significant than 1.0 g/L, the removal efficiency of phosphate did not increase. Considering economy and effectiveness, the optimal dosage was 1.0 g/L. With the increase in biochar dosage, the active sites, functional groups, and surface area of biochar [127] will gradually increase [3], which are beneficial for removing phosphorus. Wang et al. [61] found that when the dosage of biochar (Ca-pmsBC750N) was 1.5 g/L, the adsorption capacity of phosphorus reached the maximum. The removal efficiency of phosphorus by Ca-pmsBC750N increases with the increase in adsorbent dosage, reaching a removal rate as high as 96.29%. Moreover, the residual phosphorus concentration after adsorption was 0.28 mg/L, which met the first-class (A) discharge standard of municipal wastewater in China. The additional amount of biochar mainly depends on the application purpose of the biochar. If the goal is to remove phosphate from wastewater, more biochar is needed to obliterate it. If the adsorption of phosphorus by biochar is used as phosphate fertilizer, it is recommended to add less biochar [11].

In the process of nitrogen adsorption, Tan et al. [48] reported the effects of four kinds of biochars on the phosphorus removal rate and adsorption capacity. The results showed that with increasing biochar dosage (0.01 g~0.5 g), the adsorption capacity of biochar for ammonia nitrogen decreased, but the ammonia nitrogen removal rate increased. This is consistent with the research of Cheng et al. [3]. The reason may be that with increasing biochar, the active sites and functional groups of biochar gradually increase, thus increasing the removal rate. However, due to the limited initial concentration of the solution, the adsorption capacity gradually decreases with increasing amount. In addition, the research report of Wang et al. [118] also shows that with the increase in the addition amount, the adsorption amount of ammonium nitrogen decreases while the removal rate gradually increases. However, when the removal rate and adsorption capacity of ammonia nitrogen reach a certain level, further addition of biochar will reduce the removal rate and adsorption capacity, which is consistent with the research results of Hu et al. [128]. The reason may be that in the initial stage, the surface area and adsorption sites of biochar increase with the amount of biochar. If the amount of adsorbent is further increased, the surface layer of the biochar will overlap, and the active sites on the biochar will be shielded from adsorbing ammonia nitrogen [76]. Increasing the dosage of adsorbents can enhance the total specific surface area and adsorption sites of biochar, facilitating the removal of nitrogen and phosphorus from water. However, excessive addition of adsorbents can result in material wastage and potentially lead to a decrease in adsorption efficiency.

## 6. Reusability of Biochar

**Figure 14:**
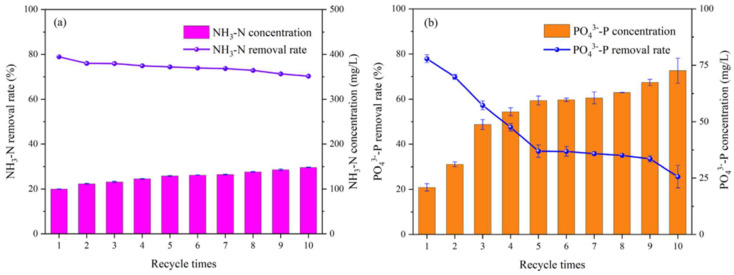
Effect of cycle times on NH_3_-N (**a**) and PO_4_^3−^-P (**b**) adsorption in pig biogas slurry by C@Mg-P. Reprinted with permission from Ref. [79]. Copyright 2023 Copyright Lintong He, Dehan Wang, Tianlang Zhu, Yongzhen Lv, Sicheng Li.

Reusability is an essential criterion for evaluating the performance of an adsorbent [71]. After the biochar adsorbs nitrogen, phosphorus, or other substances, it can be desorbed by methods such as an analyzer so that the biochar has a relatively high adsorption capacity again, and biochar with high reusability can maximize the economic and environmental benefits. The biochar (CS-MgCBC) prepared by Li et al. [123] was desorbed with ammonia-free water three times. Nevertheless, the adsorption capacity of NH_4_^+^-N decreased slightly, while the adsorption capacity of PO_4_^3−^-P was almost unaffected. Regarding the removal rate, PO_4_^3−^-P was lower than 8.6% when it was not recovered, and NH_4_^+^-N decreased by 15.7%, indicating that biochar (CS-MgCBC) is an excellent reusable adsorbent. In contrast, He et al. [79] did not desorb biochar using chemical reagents, but instead conducted pyrolysis of the biochar in a tubular furnace (at a pyrolysis temperature of 100 °C for a duration of 0.5 h) to achieve their objective. As shown in Figure 14, when the number of cycles reached 10, the removal rate of NH_3_-N in pig farm biogas slurry by biochar (C@Mg-P) slowly decreased from 78.95% to 70.31% in the tenth cycle. The removal rate was still above 70%, but the removal rate was reduced by 52.3% in 10 phosphate desorption experiments. Generally, the recycling effect of biochar (C@Mg-P) on NH_3_-N removal is excellent. On biochar loaded with P, Sun et al. [10] carried out five desorption cycle experiments, and the results showed that the removal rate of phosphorus by the biochar gradually decreased to approximately 75%. The adsorption capacity decreased from 525.4 mg/g to 375.2 mg/g, which may be caused by the decrease in effective adsorption sites in the biochar after desorption. In the desorption cycle experiment of nitrogen and phosphorus, the phosphorus removal rate decreased slightly when the analyzed biochar adsorbed nitrogen and phosphorus again. It significantly influences the nitrogen removal rate, which is consistent with the experimental results of Zhang et al. [62]. After three desorption cycles, the removal rates of NH_4_^+^-N and phosphorus by biochar ES600 decreased by 16.3% and 11.2%, respectively. Nevertheless, the removal rates remained above 70%, and the nitrogen and phosphorus adsorbed in biochar could be used to improve soil. After repeated desorption cycles, biochar still maintains a high adsorption rate for phosphorus, indicating that it has vast application potential in the field of phosphorus removal. This may be because the adsorption sites of nitrogen are destroyed more during desorption, or the adsorption sites that have adsorbed nitrogen are not entirely desorbed.

## 7. Adsorption Mechanism

There are similarities and differences in the adsorption mechanisms of nitrogen and phosphorus by biochar. Because nitrogen and phosphorus are charged in aqueous solution, they can all be removed by electrostatic attraction. Nitrogen and phosphorus can also be removed by coprecipitation. In the mechanism reported by Bian et al. [51], NH_4_^+^ and PO_4_^3−^ can form MgNH_4_PO_4_ precipitates with Mg^2+^ on the surface of biochar, and the positive charge of NH_4_^+^ can generate electrostatic attraction with the negative one carried by biochar, thus achieving the removal effect. They also analyzed the adsorption mechanism of phosphorus, using four tools, including LDH layered structure reconstruction (memory effect). However, the mechanism of phosphorus removal is not limited to this. Liang et al. [129] found that the -OH peak at 3422 cm^−1^ was enhanced due to phosphate adsorption when compared to the biochar before. FTIR phosphorus adsorption, which may be caused by the formation of hydrogen bonds, is considered to be only one of the mechanisms of phosphorus adsorption. The most important mechanism is the formation of chemical precipitation.

**Figure 15:**
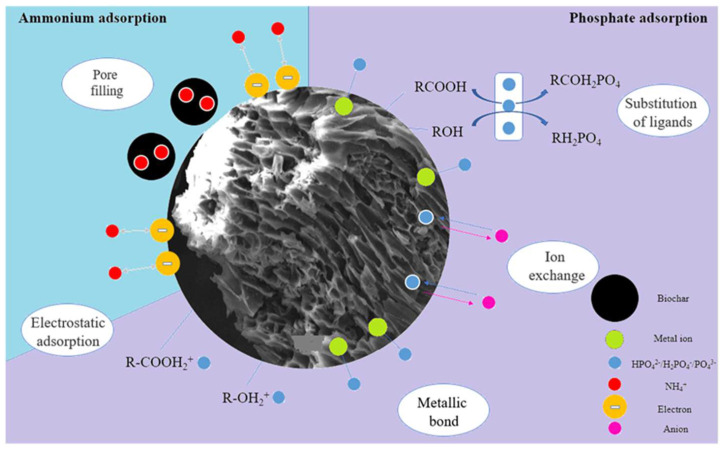
Adsorption mechanisms of ammonium and phosphate onto EBC. Reprinted with permission from Ref. [3]. Copyright 2021 Copyright Ning Cheng, Bing Wang, Qianwei Feng, Xueyang Zhang, Miao Chen.

**Figure 16:**
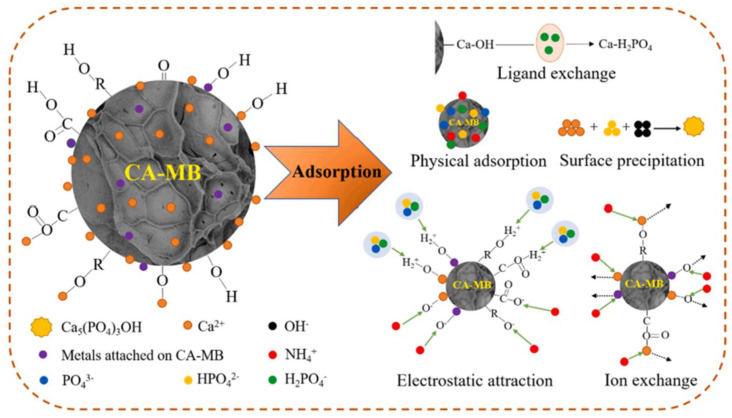
The primary adsorption mechanism of Ca-MB for phosphate and ammonium. Reprinted with permission from Ref. [125]. Copyright 2021 Copyright Qianwei Feng, Miao Chen, Pan Wu, Xueyang Zhang, Shengsen Wang, Zebin Yu, Bing Wang.

There are many pores with different sizes on the surface of biochar, which can effectively adsorb nitrogen and phosphorus. Zhao et al. [12] found that the biochar (RM/RSBC) he prepared can adsorb ammonium and phosphate through gap filling. Nevertheless, the primary removal mechanism is mainly chemical adsorption, such as surface precipitation and electrostatic attraction, which can effectively remove nitrogen and phosphorus. Some researchers also found that the kinetic model agrees with the pseudo-second-order model in phosphorus adsorption by biochar prepared by algae plants, indicating that the adsorption mechanism is chemical adsorption [130]. There are also many adsorption mechanisms in the adsorption of unmodified biochar. The zeta potential of EBC300 biochar prepared by Cheng et al. [3] showed that it is negatively charged and can effectively adsorb ammonium. In addition, during the adsorption process, phosphate ions replace the ions in EBC300, thereby increasing the adsorption capacity of phosphorus. Phosphate ions can also remove phosphorus by forming metal bonds (such as Fe-O-P and Ca-O-P) with the metal ions on the surface of EBC300. As shown in Figure 15, the mechanism of ammonium and phosphate removal by EBC300 is mainly attributed to electrostatic attraction, ligand exchange, and precipitation formation. Additionally, pore filling and ion exchange also play a certain role. Liu et al. [73] found that the adsorption process of biochar also involved ligand exchange. Through FTIR analysis before and after the adsorption of phosphate on biochar, they found that the hydroxyl group was replaced by the La-O-P surface complex, and in high pH solution, the ability of ligand exchange to remove phosphate decreased. However, the total removal rate remained at 87% because electrostatic attraction and complexation were still needed to remove phosphate. Because NH_4_^+^ is positively charged, it cannot be directly removed by precipitation with metal ions on the surface of biochar such as HPO_4_^2−^, so for the removal of ammonium, it can be electrostatically attracted with negative charges on the surface of biochar or exchanged with some ions to remove ammonium. For example, Wu et al. [131] reported that ammonium can be effectively removed by ion exchange and electrostatic attraction mechanisms in ammonium adsorption by biochar. In biochar with multiple adsorption sites, the adsorption mechanism is often prosperous. According to the report of Feng et al. [125], the biochar (CA-MB) adsorption mechanism was analyzed using various characterization methods. As shown in Figure 16, the adsorption mechanism of CA-MB for phosphate includes physical adsorption, surface precipitation, electrostatic attraction, and ligand exchange. In contrast, the adsorption mechanism for ammonium includes physical adsorption, ion exchange, and electrostatic attraction, in which both physical adsorption and electrostatic attraction can be used for ammonium. Phosphate is also removed. The capacity of removing ammonium and phosphate by each adsorption mechanism is limited, and they can be removed to greater extents by using each agent reasonably. In summary, the adsorption mechanisms for nitrogen include physical adsorption, ion exchange, electrostatic attraction, and chemical precipitation, and the adsorption mechanisms for phosphorus involve LDH layered structure reconstruction (memory effect), ion exchange, formation of inner and outer spherical complexes through functional groups on the surface, precipitation, electrostatic attraction, physical adsorption, and ligand exchange. According to the adsorption mechanism of biochar, further research is needed to remove ammonium and phosphate more efficiently.

## 8. The Resource Utilization of Biochar Loaded with Nitrogen and Phosphorus

Biochar loaded with nitrogen and phosphorus can be applied in agriculture and environmental management to achieve the multifaceted and repeated utilization of materials, with the following effects.

**Figure 17:**
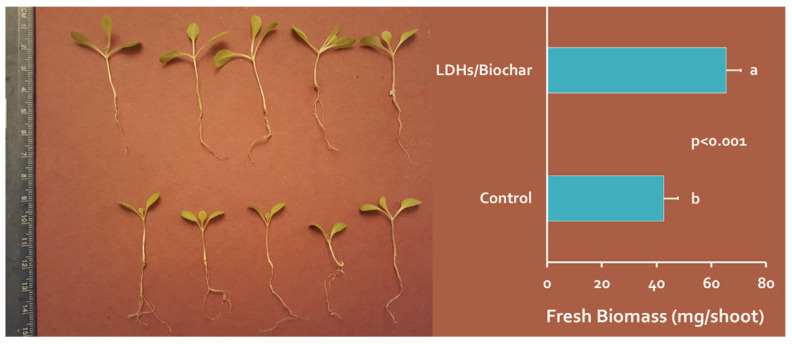
Comparison of fresh biomass of lettuce seedlings between the control group and LDHs/biochar. Reprinted with permission from Ref. [135]. Copyright 2016 Copyright Stefan Wana, Shengsen Wang, Yuncong Li, Bin Gao.

**Figure 18:**
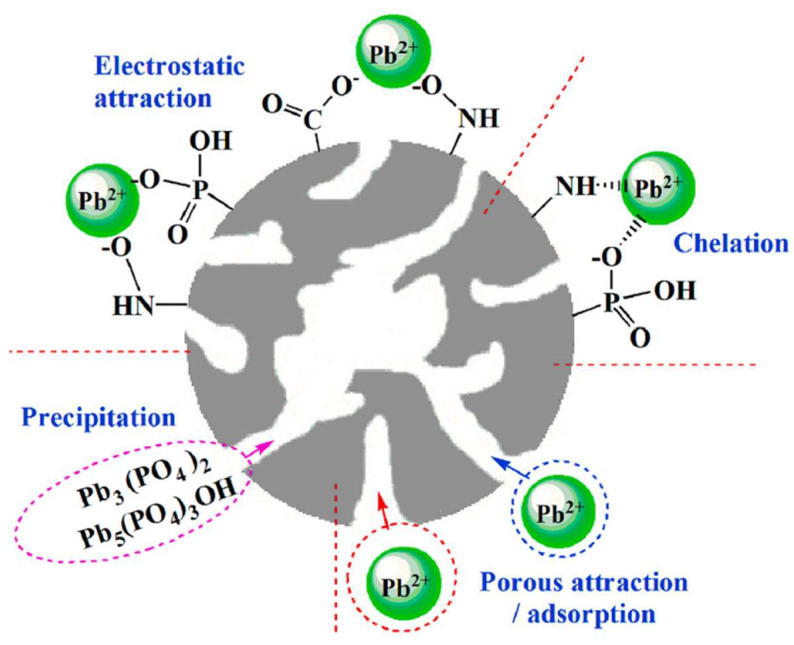
Adsorption mechanism of N and P co-doped porous biochar on Pb (II). Reprinted with permission from Ref. [137]. Copyright 2020 Copyright Jianqiu Li, Feifei He, Xiaoyang Shen, Dongwen Hu, Qiang Huang.

**Figure 19:**
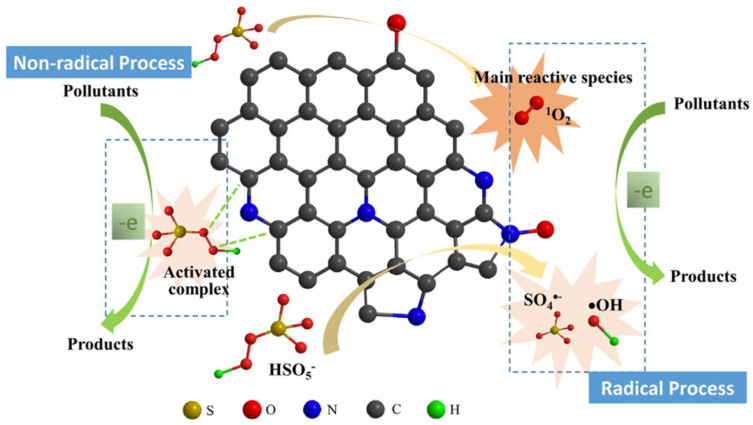
Proposed mechanism of PMS activation and MB degradation over NC-700. Reprinted with permission from Ref. [138]. Copyright 2019 Copyright Wanrong Hu, Yi Xie, Shan Lu, Panyu Li, Tonghui Xie, Yongkui Zhang, Yabo Wang.

(1) It can promote plant growth. Applying biochar loaded with nitrogen and phosphorus elements as fertilizers to plants can enhance their growth, thereby alleviating the pressure on fertilizer supply while achieving secondary utilization. Research has found that nitrogen contained in biochar can promote the growth of grass in lawns, making it a potential soil amendment [132]. In biochar loaded with phosphorus, Zhu et al. [133] discovered that biochar loaded with phosphate can enhance the growth of ryegrass, exhibiting significantly higher dry weight, growth height, and chlorophyll content [134] compared to the blank control group. Similarly, Wang et al. [135] applied biochar loaded with phosphate (LDHs/biochar) to lettuce seedlings, as shown in Figure 17. The seedlings treated with biochar loaded with phosphorus exhibited significantly higher fresh biomass and length compared to the control group. The faster growth rate of ryegrass and lettuce seedlings compared to the other groups can be mainly attributed to the ability of these two plants to absorb phosphorus elements contained in the biochar, thereby promoting their growth. 

(2) It can enhance the adsorption of heavy metal elements. Nitrogen and phosphorus co-doped biochar prepared by Fan et al. [136] exhibited excellent adsorption capacity for the heavy metal element Pd (II), with a maximum adsorption capacity of 723.6 mg/g. The adsorption mechanism is shown in Figure 18, mainly dominated by chemical adsorption. Moreover, this biochar has a high pore volume and a significant amount of nitrogen and phosphorus functional groups, which are beneficial for the adsorption of Pd (II). In addition, research has shown that the removal efficiency of Cr (VI) in water can reach more than 95% through nitrogen and phosphorus co-doped biochar, which also reduces Cr (VI) to Cr (III) [137]. The adsorption of Cr (VI) can simultaneously weaken its toxicity in the environment, highlighting the value and potential of biochar loaded with nitrogen and phosphorus in environmental remediation. 

(3) It can degrade organic pollutants. Hu et al. [138] utilized nitrogen-doped biochar (NC-700) for the removal of methylene blue (MB), achieving an adsorption capacity of 35.832 mg/g. After five cycles of adsorption, no significant loss in removal efficiency was observed, indicating good stability and reusability. After introducing peroxymonosulfate (PMS), NC-700 activated PMS, and degraded MB, a possible mechanism of PMS activation and MB degradation over NC-700 is proposed as illustrated in Figure 19. In the NC-700/PMS system, MB is mainly degraded through the free radical pathway. In addition, nitrogen-doped biochar exhibits excellent adsorption performance for metolachlor (MET) [139], toluene [140,141], acid orange 7 (AO7, anionic), and methyl blue (MB, cationic) [142]. Compared to metal-doped materials, metal-free carbonaceous materials can solve the problems of poor stability and unnecessary metal leaching, thereby preventing secondary pollution to the environment [143]. The above research indicates that biochar loaded with nitrogen and phosphorus not only promotes plant growth, but also demonstrates excellent performance and potential in adsorbing heavy metal elements and organic pollutants, thereby reducing the environmental hazards caused by pollutants.

## 9. Conclusions and Outlook

Biochar can effectively adsorb nitrogen and phosphorus in water, and the preparation method of biochar is simple, the cost is low, and the source of raw materials is broad. Biochar with adsorbed nitrogen and phosphorus can also be used in agriculture. Biochar prepared under different conditions affects the adsorption capacity of biochar for nitrogen and phosphorus by changing its characteristics. In alkaline environments, nitrogen and phosphorus adsorption is more favorable; this is because in acidic solutions, H^+^ competes with NH_4_^+^ for adsorption. At the same time, H_2_PO_4_^−^ does not easily precipitate with other metal ions, leading to decreased adsorption capacity. Regarding coexisting ions, most anions have adverse effects on phosphorus adsorption, as do cations on the adsorption of NH_4_^+^. However, it is worth noting that adding SO_4_^2−^ and Mg^2+^ at a specific concentration may increase the adsorption of nitrogen and phosphorus. Because the adsorption of nitrogen and phosphorus by biochar is an endothermic reaction (reaction enthalpy change ΔH > 0), increasing the reaction temperature can increase the adsorption capacity of nitrogen and phosphorus. Biochar adsorbs nitrogen and phosphorus through a variety of mechanisms, generally, mainly chemical adsorption, and removes nitrogen and phosphorus in the form of chemical precipitation. In future research, using each mechanism of nitrogen and phosphorus adsorption by biochar can increase the adsorption of nitrogen and phosphorus by biochar to a greater extent.

Although significant progress has been made in the adsorption of biochar, there has been limited research on the recovery of biochar in practical applications. Currently, only the recovery of biochar after adsorption of nitrogen and phosphorus can be conducted in laboratory experiments. Therefore, developing a method that can be applied to actual wastewater treatment and can recover biochar after adsorption of nitrogen and phosphorus remains challenging. In particular, in actual wastewater, there are many pollutants that compete with the target substances for adsorption, leading to a decrease in the adsorption capacity of biochar for nitrogen and phosphorus. Therefore, it is crucial to develop biochar with high ion selectivity. The excellent characteristics of biochar have attracted increasing research attention and it is considered a promising candidate for future water treatment.

## Figures and Tables

**Table 1:** Progress of nitrogen adsorption by biochar.

Raw Material	Adding Reagents or Biomass	Pyrolysis Temperature (°C)	Pyrolysis Time (h)	Name	Specific Surface Area (m^2^/g)	Pore Volume (cm^3^/g)	Model Fitting	Phosphorus Adsorption Capacity (mg/g)	Cite
Corn stalk	FeCl_3_·6H_2_O, Mg(OH)_2_	500	2	LDH@BCL	238.71	0.154	Thomas	105.73	[49]
Pig manure	MgO	550	2	MgO-PMBC	144.431	0.197	Langmuir	122.01	[50]
Corn stalk	Sodium alginate, MgCl_2_·6H_2_O, CaCl_2_			Ca/MgBC			Langmuir–Freundlich	177.25	[9]
Sludge	6H_2_O, Mg(OH)_2_	500	2	MgFe-LDH@BC	51.4556	0.0463	Pseudo-second-order	85.758	[51]
Panda manure	Fenton’s reagent, H_2_O_2_, KMnO_4_	400		FSC-400	37.61	0.13		221.7	[52]
Oak sawdust	ZnCl_2_, NaBH_4_, Carboxymethyl cellulose	600	2	nZVZ-CMC-PMBC	72.9647	0.0494	Langmuir	45.71	[53]
Oak sawdust	LaCl_3_·7H_2_O	500	0.5	La-500	10.39 ± 0.12		Langmuir	32.0	[54]
Peanut shell		400	2	C-BC400			Langmuir	25.4560	[55]

**Table 2:** Progress of phosphorus adsorption by biochar.

Raw Material	Adding Reagents or Biomass	Pyrolysis Temperature (°C)	Pyrolysis Time (h)	Name	Specific Surface Area (m^2^/g)	Pore Volume (cm^3^/g)	Isotherm	Phosphorus Adsorption Capacity (mg/g)	Cite
Coffee grounds	KOH, MgCl_2_, NaOH	500	2	MgSCG-500	107.3		Langmuir–Freundlich	112.20	[64]
Sheep manure	Tested oyster	800	2	BC-Ca5	5.09	0.04	Langmuir	79.33	[65]
Sheep manure, Tested oyster shells	LaCl_3_·7H_2_O	800	2	BC-La4	12.04	0.06	Langmuir	92.67	[65]
Yellow Pine wood	Ca(OH)_2_	100	2	Ca-BC100	120.26		Langmuir	125.60	[66]
Reed straw	FeSO_4_·7H_2_O, NaBH_4_	700	2	Fe-700-BC	53.25		Langmuir	95.20	[67]
Wheat straw	LaCl_3_·7H_2_O, Na_2_CO_3_	300	2	LCB300				64.30	[68]
Wheat straw	LaCl_3_·7H_2_O, NaOH	800	2	LHB800				65.00	[68]
Corn cob	FeCl_3_·6H_2_O, DETA	900	1	ZVI/BC-N	379.63	0.31	Freundlich	82.78	[69]
Corn cob	FeCl_3_·6H_2_O	900	1	ZVI/BC	215.33	0.14	Langmuir	17.93	[69]
Banana straw	MgCl_2_	430	4	BSB	6.6839	0.0267	Langmuir	31.15	[70]
Corn stover	CaCl_2_	800	1	CaBC800	0.02383	0.1326	Langmuir	33.944	[71]
Bagasse powder	Marble waste	800	2	Mar-BC800	92.81	265,000	Langmuir	263.17	[72]
Bagasse powder	Calcium-rich sepiolite	800	2	Sep-BC800	106.67	397,000	Langmuir	128.21	[72]
Canna	La(NO_3_)_3_·6H_2_O	800	1	CBC-La			Langmuir	37.37	[73]
Canna		800	1	CBC	193.1461	0.1228	Langmuir	9.47	[73]

**Table 3:** Comparison of nitrogen adsorption between the original biochar and modified biochar.

Raw Material	Modification Reagent	Pyrolysis Temperature (°C)	Pyrolysis Time (h)	Name	Specific Surface Area (m^2^/g)	Total Pore Volume (cm^3^/g)	Model Fitting	Nitrogen Adsorption Capacity (mg/g)	Cite
Walnut shell		600	2	BC	285.4958	0.1156	Sips	60.82	[47]
Walnut shell	C_2_H_4_N_4_	600	2	N@BC	456.2524	0.3425	Sips	73.51	[47]
Walnut shell	FeCl_3_·6H_2_O	600	2	Fe@BC	536.4587	0.3625	Sips	83.42	[47]
Walnut shell	C_2_H_4_N_4_, FeCl_3_·6H_2_O	600	2	Fe/N@BC	967.1084	0.7425	Sips	111.87	[47]
Maize straw		450	2	BC	162.50	0.20	Langmuir	10.379	[48]
Maize straw	MgCl_2_	450	2	MBC	152.00	0.31	Langmuir	18.335	[48]
Oak sawdust		500	0.5	CK-300	0.050 ± 0.02		Langmuir	5.31	[54]
Oak sawdust	LaCl_3_·7H_2_O	500	0.5	La-300	1.57 ± 0.12		Langmuir	10.1	[54]
Peanut shell		500	2	BC			Sips	3.83	[95]
Peanut shell	KMnO_4_, KOH	500	2	MBC			Sips	6.92	[95]

**Table 4:** Comparison of phosphorus adsorption between original biochar and modified biochar.

Raw Material	Modification Reagent	Pyrolysis Temperature (°C)	Pyrolysis Time (h)	Name	Specific Surface Area (m^2^/g)	Total Pore Volume (cm^3^/g)	Isotherm	Phosphorus Adsorption Capacity (mg/g)	Cite
Oak sawdust		500	0.5	CK-500	7.72 ± 0.19		Langmuir	142.7	[54]
Oak sawdust	LaCl_3_·7H_2_O	500	0.5	La-500	10.39 ± 0.12		Langmuir	32.0	[54]
Yellow Pine wood		500	2	BC	260.50			4.00	[66]
Yellow Pine wood	Ca(OH)_2_	500	2	Ca-BC100	120.26			138.70	[66]
Canna		800	1	CBC	193.1461	0.1228	Langmuir	9.47	[73]
Canna	La(OH)_3_	800	1	CBC-La			Langmuir	37.37	[73]
Mimosa pudica		500	2	BC	285.53	0.15		5.1	[84]
Mimosa pudica	AlCl_3_·6H_2_O	500	2	BAl1	130.48	0.12		65.6	[84]
Mimosa pudica	AlCl_3_·6H_2_O	500	2	BAl2	255.85	0.28		70.6	[84]
Wheat straw		600	2	BC	227.12		Langmuir	1.64	[96]
Wheat straw	MgCl_2_, AlCl_3_	600	2	MABC	268.50		Langmuir	153.40	[96]
Flour		600	2	BC			Langmuir	48.44	[97]
Flour	Ca(OH)_2_	600	2	Ca-BC (2:1)			Langmuir	314.22	[97]

## Data Availability

No new data were created or analyzed in this study. Data sharing is not applicable to this article.
